# Catastrophic hemorrhage of adrenal pheochromocytoma following thrombolysis for acute myocardial infarction: case report and literature review

**DOI:** 10.1186/1749-7922-9-50

**Published:** 2014-09-20

**Authors:** Tarik Souiki, Zoheir Tekni, Hind Laachach, Amal Bennani, Youssef Zrihni, Azeddine Tadmori, Farida Ajdi, Abderahim Bouazzaoui, Laila Chbani, Hafid Akoudad, Khalid Mazaz, Said Aitlaalim

**Affiliations:** School of medicine and pharmacy of Fez, Sidi Mohammed Ben Abdellah University, BP: 1893, km 2.200, route de Sidi Hrazem, Fez, 30000 Morocco; Department of surgery, University Hospital Hassan II, BP: 1893, km 2.200, route de Sidi Hrazem, Fez, 30000 Morocco; Department of anesthesiology, University Hospital Hassan II, BP: 1893, km 2.200, route de Sidi Hrazem, Fez, 30000 Morocco; Department of Cardiology, University Hospital Hassan II, BP: 1893, km 2.200, route de Sidi Hrazem, Fez, 30000 Morocco; Department of pathology, University Hospital Hassan II, BP: 1893, km 2.200, route de Sidi Hrazem, Fez, 30000 Morocco; Department of endocrinology, University Hospital Hassan II, BP: 1893, km 2.200, route de Sidi Hrazem, Fez, 30000 Morocco

**Keywords:** Hemorrhage, Pheochromocytoma, Thrombolysis, Hémorragie, Phéochromocytome, Thrombolyse

## Abstract

We describe here the case of a 62-year-old man with acute abdominal syndrome and severe hemorrhagic shock following successful thrombolysis for acute cardiac infarction. Emergency surgical exploration revealed extensive intraperitoneal and retroperitoneal hemorrhage resulting from the rupture of a large adrenal tumor. The diagnosis of pheochromocytoma was confirmed by histological findings. The patient died a few hours after surgery from multiorgan failure despite resuscitation attempts. This report discusses the diagnosis difficulties, treatment approach, and relevant literature.

## Abstract (fr)

Nous rapportons l’observation d’un patient âgé de 62 ans, qui présente un syndrome abdominal douloureux aigu associé à un choc hémorragique sévère au décours d’une thrombolyse pour infarctus du myocarde. L’exploration chirurgicale urgente objective une hémorragie extensive retro- et intra péritonéale secondaire à une rupture d’une masse surrénalienne. Le diagnostic de phéochromocytome a été confirmé par l’examen histologique de la pièce opératoire. L’évolution était rapidement fatale, aboutissant au décès du patient par défaillance multi-viscérale quelques heures en postopératoire. Seront discutés à travers cette observation et une revue de la littérature, les difficultés diagnostiques et de prise en charge de cette redoutable complication.

## Introduction

Pheochromocytoma presenting with acute hemorrhage is extremely rare. This report highlights a case of fatal retroperitoneal and intraperitoneal hemorrhage in a patient with undiagnosed pheochromocytoma who had been thrombolyzed for an acute myocardial infarction. To our knowledge, this is the first case of acute hemorrhage into pheochromocytoma associated with thrombolysis to be reported in the literature.

## Case report

**Figure 1:**
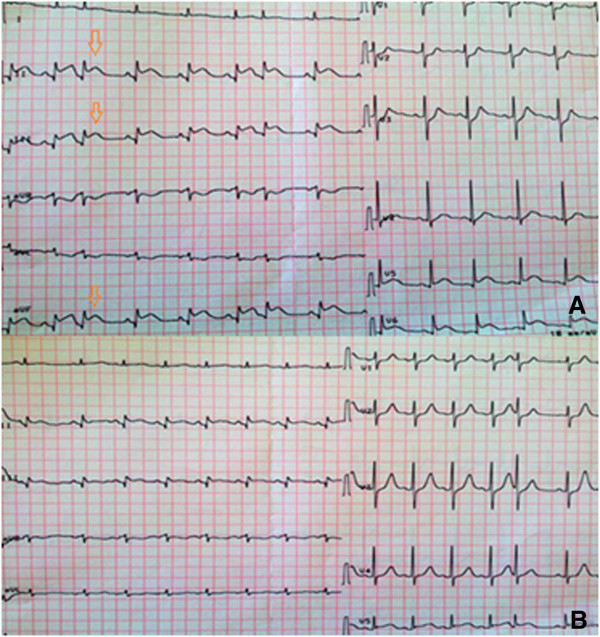
**Electrocardiogram’s data. (A)** ST-segment elevation (arrow) at admission. **(B)** ST-segment resolution after thrombolysis.

**Figure 2:**
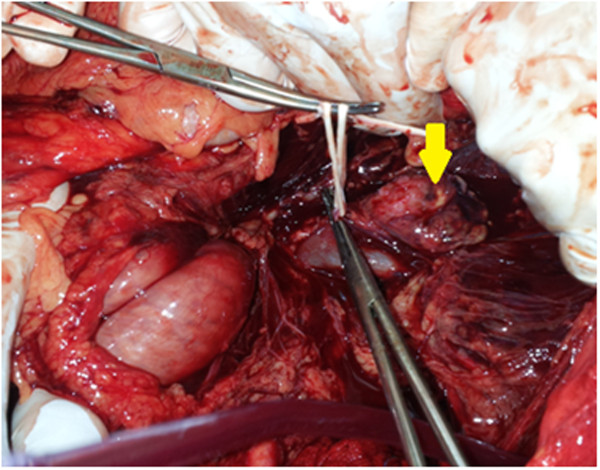
**Per-operative view showing adrenal tumor (arrow) with extensive retroperitoneal hemorrhage.**

**Figure 3:**
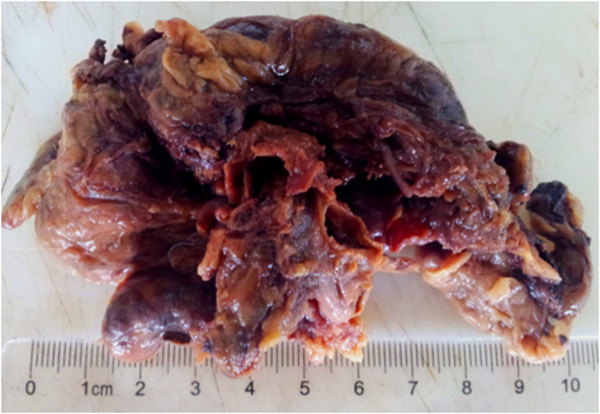
**Macroscopic inspection of the resected adrenal pheochromocytoma( (10 × 7 × 2 cm).**

**Figure 4:**
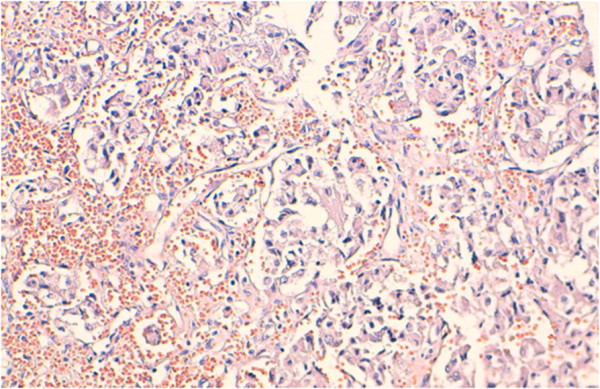
**Histological findings of the tumor.** Pheochromocytoma cells located around fine vascularisation (hematoxylin-eosin, X20).

A 62-year-old man with a medical history of diabetes mellitus and smoking was admitted to the cardiology unit with acute chest pain. The electrocardiogram showed ST-segment elevation in the inferior leads aVF, D2, and D3 (Figure 1A). The laboratory tests indicated enzymatic activity typical of myocardial necrosis: elevation of troponine and creatine kinase. There were no abnormalities in other routine blood tests. Echocardiography revealed a normal-sized heart with good left ventricular contractility. Therefore, the diagnosis of uncomplicated acute inferior myocardial infarction was established. Our patient was given oxygen, electrocardiographic (ECG) monitoring, oral aspirin, clopidogrel, simvastatine, and a subcutaneous injection of low-molecular weight heparin. In the absence of contraindications, intravenous thrombolytic therapy with a single bolus of tenecteplase (metalyse®) was administered, and a significant resolution of segment ST elevation was obtained 30 minutes later (Figure 1B). In the following hour, after this "successful" thrombolytic therapy, it was noted that our patient complained of sudden abdominal pain. On examination, he was pale and sweating. He had tachycardia and his blood pressure (BP) had rapidly dropped to 70/30 mmHg. The abdomen was tender and distended. Given these symptoms of hypovolemic shock, resuscitation with fluid repletion and transfusion were rapidly initiated. A bedside ultrasound abdominal examination, although constrained by reflex ileus, showed blood effusion in the free abdominal cavity. Before we were able to perform abdominal scanning to characterize the source of this intra-abdominal hemorrhage, the patient’s hemodynamic status began to deteriorate. Despite the infusion of blood pressure-raising drugs, the patient had rapidly recurring hemodynamic instability, which obliged us to perform an emergency laparotomy for suspected intra-abdominal bleeding. Surgical exploration revealed extensive retroperitoneal and intraperitoneal hemorrhage due to the rupture of a large left adrenal tumor (Figures 2 and 3). A left adrenalectomy was performed, and hemostasis was obtained. During the tumor manipulation, we observed that our patient presented a hypertensive peak of 22/12 mmHg, which was managed with nicardipine infusion. In contrast, the patient’s BP dropped rapidly after the complete excision of the tumor. Given this labile perioperative BP, a ruptured pheochromocytoma was suspected. This diagnosis was later confirmed by the final histological examination (Figure 4). The patient was transferred to the intensive care unit and died from multiorgan failure several hours after surgery, despite resuscitation attempts.

## Discussion

Pheochromocytomas are catecholamine-secreting neuroendocrine tumors that arise from chromaffin cells of the adrenal medulla [1]. The typical symptom is persistent or paroxysmal hypertension. Of note, it is estimated that pheochromocytomas are present in 0.1-0.6% of patients with hypertension [2]. Other presenting features include palpitations, headache, sweating, pallor, tremors, and anxiety [3]. However, 10-30% of pheochromocytomas are asymptomatic and inadvertently discovered in imaging performed for other reasons [4]. Acute hemorrhagic rupture as an initial manifestation of pheochromocytoma is an extremely rare entity.

A brief review of the literature reveals 65 cases of acute ruptured pheochromocytomas from its first description by Cahil in 1944 [5] until present [6–20]. The clinical characteristics of the reviewed cases are shown in Table 1. In most cases, the hemorrhage was contained in the retroperitoneum. Nevertheless, bleeding was extensive in approximately 20% of reported cases and concerned both the retroperitoneal and free peritoneal cavity as in our case [6–20]. The rupture was usually spontaneous and less than one-third of patients indicated a previous history of suggestive symptoms of pheochromocytoma [6, 13]. The mechanism is unclear, but it is presumed to be due to increased intratumoral intravascular pressure that may be precipitated by paroxysms of hypertension or necrosis [17, 21]. Abdominal trauma and medications such as anticoagulants or alpha-blockers were also mentioned by several authors as possible initiating factors of rupture [10, 22–24]. Concerning thrombolysis, although that its use is recognized as being associated with an increased bleeding risk [25], ours is the first case, to our knowledge, to suggest an association between hemorrhaging pheochromocytoma and thrombolytic therapy.

**Table 1:** **Clinical characteristics of 65 patients with ruptured pheochromocytoma**

Patients	n = 65
**Period of publication**	
** - Before 1985**	26
** - 1985 or later**	39
**Median age**	50 (15–80)
**Gender**	37/28 (1,3) (57%)
**Tumor side**	
** - Right,**	31
** - Left,**	31
** - Bilateral**	3
**Symptoms**	
** - Acute abdomen**	50 (77%)
** - Shock**	37 (57%)
** - Lumbar pain**	16 (25%)
** - Chest pain**	10 (15%)
**Surgical setting**	
** - Emergency**	35 (54%)
** - Delayed surgery**	21 (32%)
** - Not performed**	9 (14%)
**Death/Survival**	18/65 (28%)

The reviewed patients ranged in age from 15 to 80 years, with a mean age of 50; 57% of patients were men. Pheochromocytomas were right-sided in 48% of cases, left-sided in 48%, and bilateral in 4%. The median lesion size was approximately 7 cm [19]. In most cases, patients presented with an acute onset of abdominal pain. Signs of severe peripheral vasoconstriction (cold, sweating, and pale extremities) due to the release of catecholamine were usually associated [14]. Occasionally, other symptoms like chest pain or lumbar pain were seen in 15% and 25% of cases, respectively [6–20]. Rarely, as in the case of our patient, was the clinical presentation heavily dominated by severe shock. Indeed, the shock status was considered by several authors to relate to either hemorrhagic shock or a sudden drop in the blood catecholamine level due to tumor necrosis [6]. Indeed, obtaining a diagnosis under such emergency settings is extremely challenging. Thus, among the 50 cases of ruptured pheochromocytoma reviewed by Kobyashi et al. in 2005, the rate of preoperative misdiagnosis was 60% [6]. According to this study, there is a strong correlation between hemodynamic instability and misdiagnosis. In reality, hemodynamic instability often obliges the surgeon to perform emergency surgery without sufficient investigations, which is significantly associated with a high mortality rate. In our case, having an accurate preoperative diagnosis was rendered more difficult because of the initial presentation of cardiac infarction, and as a result, any sign would first be attributed to cardiac disorders, which was a source of great delay in the diagnosis.

If we analyze causal relationship between pheochromocytoma and cardiac infarction in our case, two hypothetic scenarios arise. First, the pheochromocytoma was spontaneously ruptured and induced cardiac disorders that simulated a cardiac infarction. The mechanism in this case, as described in a few published cases illustrating cardiac involvement in pheochromocytoma [26], may be secondary to the cardiac toxicity of catecholamine or to a coronary spasm. In such cases, coronary angiography, when performed, demonstrated no significant coronary artery disease despite the ST-elevation on the electrocardiogram. The second eventuality is that our patient with an underlying history of cardiovascular factors (diabetes mellitus and smoking) had a real cardiac infarction, leading to bleeding of the silent pre-existing pheochromocytoma through increased intratumoral stress [10, 26]. In both cases, it is clear that thrombolytic therapy administered in this context may play role in deteriorating bleeding.

Whatever the clinical picture in the foreground, computed tomography (CT) scanning of the abdomen is still the most useful examination for diagnosis [27]. It showed a heterogeneous mass and hematoma in the suprarenal region of retro-peritoneum [6]. CT also allows the adrenal mass to be distinguished from adjacent structures, particularly a ruptured aortic aneurysm, which is the major differential diagnosis to consider [6, 7]. Nevertheless, CT is not always feasible in emergency conditions as with our patient. In fact, it was performed in 31 (79%) of the 39 cases reported since 1985, when this type of examination began to be widely used in clinical settings. An ultrasound abdominal examination, more accessible in acute settings, can show blood infusion in the retroperitoneum or in the free peritoneal cavity, but it is still generally unhelpful for diagnosing the nature of the retroperitoneal hemorrhagic mass [7]. Concerning endocrinological tests, these are generally not useful in an acute setting; because they cannot provide results in time to be of any assistance [7].

When requiring an accurate diagnosis, the optimal management is based on the appropriate medical preparation before any surgical procedure. This should include adequate alpha-adrenergic blockade, infusion therapy, blood resuscitation, and correction of coagulopathy [15]. These medical measures and the time of surgery are the decisive factors in management and outcome. In fact, surgery in acute settings is recognized to carry a high risk of morbimortality [6]. This is even more evident when performed without medical preparation. Thus, in one series of 50 patients [6], there were no deaths among 12 patients who underwent delayed elective surgery with medical preparation, while 17 (45%) of the 38 patients who underwent an emergency surgical procedure, without appropriate medical preparation, died [6]. Furthermore, delayed surgery has other advantages. First, it allows the patient to recover from the acute event and benefit from an a-adrenergic blockade before surgery [16]. Second, it permits surgical removal under laparoscopy after the resolution of the hematoma and the inflammatory response. Third, it allows an endocrinological test to be performed to discriminate the pheochromocytoma from other bleeding adrenal tumors. Thus, according to a review of 133 cases of hemorrhaging adrenal mass published by Marti et al., other differential diagnoses of pheochromocytoma representing 48% of the reviewed cases were metastatic lesions (14%), hematoma (13%), myelolipoma (10%), adrenocortical carcinoma (7%), adenoma (4%), pseudocyst/hematoma in pregnancy (4%), and lipoma (1%) [19]. In light of these considerations, we believe that delaying surgical procedure must be the main aim of any medical management, while emergency intervention must be restricted to patients who have not been responsive to maximal medical resuscitation.

The mortality rate as observed in the literature was 28% (18/65), with death often resulting from bleeding, heart failure from excessive catecholamine, or postoperative severe hypotension or pulmonary edema [6]. In an effort to better define the prognosis predictors of mortality, Kobyashi et al. performed a prognosis analysis [6]. According to this study, hemodynamic instability and misdiagnosis are the powerful predictors of mortality [6].

Recently, to avoid emergency surgery in patients with ongoing bleeding, the use of transarterial embolization (TAE) was increasingly reported as being a safe alternative to achieve hemostasis and maintaining the patient in good condition until elective surgery can be performed [9, 13, 16, 17, 21, 28]. In fact, this interventional technique is not novel in pheochromocytoma and it has been used to manage malignant hypertensive crisis and palliate hyperfunctioning malignant tumors [29, 30]. In our literature review, six cases describing the use of TAE in managing ruptured pheochromocytoma were identified [9, 13, 16, 17, 21, 28]; the clinical details are summarized in Table 2. The outcome was remarkably excellent with no deaths reported among all patients. Nevertheless, interventional capabilities may not always be available in emergency conditions, and an unstable patient, particularly with intraperitoneal hemorrhage, may require emergency surgery as in our case. For this reason, we believe, as several authors have suggested, that it is reasonable to consider the diagnosis of ruptured pheochromocytoma in the differential diagnosis of any patient with an acute abdominal syndrome. This eventuality must also be kept in consideration whenever surgeons and anesthetists are faced with emergent intra-abdominal hemorrhage with unknown preoperative diagnosis.

**Table 2:** **Clinical characteristics of 6 patients managed by TAE prior to surgery**

	Year	Patient	Symptoms	Tumor side	Timing of delayed surgery	Outcome
**Ito** [28]	1997	68 F	Abdominal pain schock	Left	3,5 months	Survival
**Park** [21]	2003	32 M	Abdominal pain	Right	3 weeks	Survival
**Pua** [9]	2008	67 M	Abdominal pain	Right	2 moths	Survival
**Habib** [13]	2010	42 M	Adbominal pain with palpitation	Right	1 month	Survival
**O'neal** [16]	2012	38 M	Abddominal pain + schock	Bilateral	5 months	Survival
**Kumar** [17]	2013	63 M	Loin pain + schock	Left	1 month	Survival

## Conclusion

While a rare event, ruptured pheochromocytoma continues to be highly lethal in modern times. Usually spontaneous, this accident may also be initiated by trauma or medication. Thrombolytic therapy as described in the current case may play a role in accelerating bleeding. Proceeding to surgery with an occult pheochromocytoma, however, could be catastrophic. For this reason, a high index of suspicion is required for preoperative accurate diagnosis, which is a crucial step for adequate management. Computed scanning of the abdomen is the most useful examination for diagnosis, while medical preparation followed by delayed elective surgery is the optimal treatment. Alternatively, TAE may be employed to stop bleeding in an instable patient in order to avoid emergency surgery associated with a high mortality rate.

## Consent

Written informed consent was obtained from the patient’s relatives for the publication of this report and any accompanying images.

## Abbreviations

BP: Blood pressure
CT: Computed tomography
ECG: Electrocardiography
TAE: Transarterial embolization.
